# Effects of Pandemic Outbreak on Economies: Evidence From Business History Context

**DOI:** 10.3389/fpubh.2021.632043

**Published:** 2021-03-12

**Authors:** Yunfeng Shang, Haiwei Li, Ren Zhang

**Affiliations:** ^1^Zhejiang Yuexiu University of Foreign Languages, Shaoxing, China; ^2^Tianjin University of Commerce, Tianjin, China; ^3^Texas State University, San Marcos, TX, United States

**Keywords:** coronavirus, pandemic outbreak, business history, LME economies, CME economies

## Abstract

The coronavirus pandemic has highlighted the capitalist dysfunction showing that considering profit over people can be deadly. The study reveals the LME economies were more responsive toward the impact of the disease outbreaks as compared to the CME economies wherein the impact of the disease was moderated by the government involvement. This allows us to draw that the impact of the disease outbreaks can be moderated by increasing the involvement of the government authorities.

## Introduction

Infectious diseases are one of the major causes of death responsible for the quarter to one-third of the mortality worldwide. Despite major developments in the pharmaceutical industry, the spread of infectious diseases is rising due to globalization, increased travel and trade, urbanization, populated cities, changes in human behavior, reviving pathogens and improper use of antibiotics (1). The recent virus outbreak Covid-19 shows that infectious diseases spread easily due to open economies and easily threaten nations' economic stability. Previous infections such as Black Death, SARS, Influenza H1N1, and Swine Flu had caused similar economic impacts worldwide. Covid-19 is more contagious, and its ability to sustain on surfaces makes it more challenging to curb. It is considered more contagious than influenza and swine flu as it transmits between people easily. The second feature is the delay in developing treatment drugs and their approval because the initial infection causes significant mortality and damage to the economy. Another feature of Covid-19 is the constant evolution and resistance of microbes toward antibacterial agents, making them a continuous and recurring threat. The majority of the outbreaks are recurring, and the current Covid-19 outbreak may evolve and recur as it is considered to be the second strain of severe acute respiratory syndrome (SARS-CoV-2), the first strain of which occurred in 2002–03 (SARS-CoV-1) (2).

The coronavirus pandemic has highlighted the capitalist dysfunction, which is considered to be partly based on the priority given to profit rather than people's need. Pharmaceutical companies would have started developing the vaccine for coronavirus a long time ago if the society had not been capitalist. The novel coronavirus spreading fast around the world belongs to the family of coronavirus (SARS and MERS) that are already familiar to us for a long time. It could have been possible to begin search for coronavirus vaccine and treatments long time ago so that the most recent outbreak of coronavirus could be prevented to some extent. But pharmaceutical companies did not initiate this research because the treatment did not seem to be profitable enough (3). It takes 12–18 months for researchers to develop vaccine to fight against Covid-19. As per the epidemiologists, coronavirus could kill up to 50 million people worldwide (4). Many of these deaths could have been avoided if the vaccine was introduced. And the vaccine has not been developed as it was not profitable for pharmaceutical companies.

The rest of the paper is organized as follows. Section A General Equilibrium Approach discusses a general equilibrium approach to the impacts of pandemic outbreak and provides brief literature review. Section Methodology and Empirical Model explains the methodology and empirical model. Section Empirical Findings discusses the empirical findings. Section Conclusion concludes.

## A General Equilibrium Approach

The characteristics of coronavirus pandemic can be analyzed through consistent characteristics of historical pandemic outbreaks. The economic effects of pandemics have been analyzed in the existing studies. A partial equilibrium approach is only focused on the health sector and the forgone earnings due to the mortality from the disease. It ignores its impact on other sectors and the other parts of the country. It is, therefore, perceived as an incomplete approach. We applied a general equilibrium approach to the economic impact of pandemic outbreaks and health diseases as the equilibrium approach is an appropriate method of comprehensively study the consequences. Under a general equilibrium approach, the health, economic and social impacts of the pandemic can be analyzed.

### Health Impacts

The health impacts of pandemics are disastrous. During the Black Death pandemic, ~30–50% of the population of Europe wiped out. In the 1980s, 35 million people died due to HIV, AIDS, and Ebola in 2014, which caused 10,600 deaths in Guinea, Sierra Leone, and Liberia in West Africa (5). Pandemic affects the young and economically active population disproportionately. The morbidity and mortality rates are higher for younger people as they tend to have lower immunity than the older generation. Thus, the pandemic's major impact is that it causes a significant increase in the years of life lost.

Moreover, many infectious diseases have lifelong consequences, and it can become more severe in pandemics. For example, the medication of Zika virus has life-long chronic effects on the health of the patient. Pandemics' indirect effects on health include the depletion of resources for routine healthcare and decreased childhood immunization rates, and reduced healthcare access due to the inability to travel. During the influenza pandemic in 2009, a surge in hospital admissions due to influenza and pneumonia caused an increase in deaths due to stroke and heart attack. Therefore, it is difficult to distinguish between the deaths attributable to the pandemic and other unassociated diseases that are merely coincidental. Healthcare workers' ability to provide care is also reduced as they fall ill themselves, are required to take care of family members or children, or even the fear of catching the disease also makes them receptive.

### Economic Impacts

Pandemics cause a short-term fiscal impact and a long-term economic impact on the nations around the world. Efforts to curb the pandemic include imposing quarantine, preparing health facilities, isolating infectious cases, and tracing contacts involving public health resources, human resources and implementation costs. It also involves health system expenditures to provide health facilities to infectious cases and the arrangement of consumables such as antibiotics, medical supplies, and personal protective equipment.

Pandemics can also result in declined tax revenues and increased expenditure, which causes fiscal stress, especially in lower-middle-income countries (LMICs) where fiscal constraints are higher, and tax systems still need improvement. This economic impact severity was observed during the Ebola virus in Liberia due to the rise in public health expenditure, economic downfall, and revenue decline due to the government's inability to raise revenue because of quarantine and curfews. Economic shocks are common during pandemics due to shortage of labor because of illness, rise in mortality, and a fear-induced behavior. Other than labor shortages, disruption of transportation, closed down of workplaces, restricted trade and travel, and closed land border are reasons for the pandemic's economic slowdown.

### Social and Political Impacts

Pandemics have significant social and political impacts such as clashes between nations, population displacement, and increased social tension and discrimination. Many pre-modern pandemics have caused serious demographic shifts, morality shocks, and social and political disturbance. Empirical evidence suggests that pandemics can create political tensions and unrest, especially in nations with weak institutions. The 2014 Ebola virus resulted in political and social unrest in the state as government-imposed quarantine and curfews to mitigate the disease's spread with security forces that the general public perceived as a conspiracy and opposing the government. This issue caused riots and violence in the country, involving threats to health care personnel and damaging healthcare facilities and supplies. Modern pandemics have subtle social disruptions such as anxiety, social isolation, fear-inducing behavior, and economic hardships.

### Varieties of Capitalism and Business History

The “Varieties of Capitalism” framework designed by Peter Hall and David Soskice has become a benchmark in the political literature on advanced industrial economies. The framework (6) discusses two capitalist arrangements: coordinated market economies (CME) and liberal market economies (LME). This framework also suggests that market pressures, such as globalization and industrial pressures, will ultimately lead to the convergence of the most efficient capitalism form. The idea of the “Varieties of Capitalism” framework is that although both the models represent different capitalism arrangements, yet both are durable and logical even in the challenging industrial environment. Many people who are supportive of egalitarian capitalism believe that the coordinated market economies (CME) are on the verge of a breakdown. This theory of “Varieties of Capitalism” is influential and provides reassurance to the people who are concerned about the breakdown of institutions characteristics.

The varieties model of capitalism has also been criticized by scholars for its overemphasizing on the flexibility of capitalism models for the reason that many countries define their institutional arrangement, especially when the coordinated market economies are under pressure and facing reforms. The critics of the framework believe that the economies are shifting toward liberalization and this pervasive shift negates the basic explanation of this model. By undermining the differentiation, it portrays between coordinated market economies and liberal market economies (7). The supporters of varieties framework typically respond by defending the differentiation of liberal and coordinated market economies. The varieties framework is, thus, an inconclusive debate among supporters and critics. For companies to succeed in this globalized economy, state support is essential either directly or implicitly. Market economies such as Japan and South Korea have experienced capitalist maturity, that is, businesses have experience of investment, technological and managerial decisions and the support of government have helped them make space in the foreign markets and succeed (8). Thus, coordination plays an important role, but the participation of association is highly essential.

For this study, we consider UK, Australia, Canada, and New Zealand representing liberal market economies (LME) and Japan, Germany, and Sweden representing coordinated market economies (CME).

### Covid-19 and Global Development

Covid-19 highlights the need to understand contemporary global challenges rather than focus on a narrower international development approach. The international development paradigm focuses on bilateral relations based on aids provided to each other, while a global development approach discovers the processes and issues related to the countries. Global international development focused on joint problems and shared issues such as global warming, terrorism, pandemics, etc. Global development is concerned with recognizing that an equitable world is formed through cooperation and shared values rather than just transforming a developing economy to a developed one. The global development paradigm is based on three important aspects. First, the interrelationship between contemporary capitalist countries goes beyond the national boundaries (9). Second, there are several challenges that nations all around the world are facing together. Third, global development is about helping each other deal with the common challenges and reduce global inequality. These goals have been recognized and part of the global Sustainable Development Goals (SDGs) and other agreements and treaties. Covid-19 makes it an urgency to use a global development approach for dealing with the common problems and challenges. The interconnected world has led to the spread of COVID-19 in a very short time. Indeed, it is a good example of the countries' common challenges and the global public good's failure. The pandemic has caused distressing economic, health and social impacts worldwide (10). The impact of COVID-19 cannot be only assessed in economic terms. It had devastating mortality and fatal rates across the United States and European countries in the North. China, Brazil, Mexico, Africa, and other Southern countries also had high infectious rates.

Apart from the health impacts, the pandemic has created the worst social and economic impact on humans' lives (11). There has been a loss of employment and livelihood, and people suffer from anxiety due to social contacts' loss. The global development paradigm's importance can be examined by assessing the impact of the Covid-19 pandemic across global value chains, debt, and digitalization.

#### Global Value Chains

Covid-19 has severely impacted the global value chains across the globe, especially the agricultural and industrial forms over the past 30 years (12). The pandemic caused a serious shortage in supplies of goods manufactured in China, especially the shortage of medical supplies affected many countries' health scenario. Due to growing nationalism and protectionism for industrial sovereignty, many countries have imposed export ban, which resulted in the shortage of medical supplies such as pharmaceutical drugs, personal protective equipment (PPE kits), and other medical products. As a result, the pressure on domestic value chains has increased, and de-globalization has emerged again. The value chains will have to be restructured after the pandemic to improve the quality and quantity of jobs and ensure sustainable transitions.

#### Debt

Public finances have been negatively affected by the Covid-19 pandemic. The closing of economies and reduced lending opportunities has decreased the value of local currencies making repayment of dollar-denominated debt harder (13). Governments are also facing a fiscal deficit due to increased social protection expenditure for the unemployed and poor and reduced tax revenues. The debt from Covid-19 is different from that of the financial crisis of the 1980s or 2007–08 and will not be explainable through the international development paradigm (14).

#### Digitalization

A positive impact of COVID-19 on third world economies has been the increase in digitalization. With the increasing threat of infection transmission through physical contact, the virtual space of transactions has gained popularity (15). The chance of its spread through social contact has accelerated online working platforms and digitally organized logistics. With online transactions and digital platforms for work, there is an opportunity to develop a centralized database that can serve as an economic asset. It has become essential to be a part of the global digital drive for improved socio-economic fortunes and mitigate the impact of the Covid-19 pandemic through digitalization.

Thus, Covid-19 requires a global development perspective rather than an international development paradigm as a global development paradigm can effectively confront the challenges. It prioritizes the collaboration on a global level rather than focusing on national and state issues as the countries' problems require a foresighted approach.

## Methodology and Empirical Model

The study examines the trend in capitalist economies during times of pandemic with a business history perspective. Both qualitative and quantitative analysis would be conducted to assess the economic consequences on Coordinated Market economies (CME) and Liberal Market Economies (LME).

### Quantitative Analysis

The study uses Computable General Equilibrium (CGE) to model the pandemic outbreak's economic implications on capitalist economies. CGE model involves using actual economic data to assess the changes in an economy as a reaction to change in external factors such as technological change, policy change, etc. It works on the assumption that countries have no barriers and are constantly engaged in trade with perfectly competitive markets and homogenous technologies. The model discusses economic activity in all countries as a function of world economic output. The economic output involves using five major inputs—capital, skilled and unskilled labor, natural resources and land.

Our research considers labor and capital as major functions of output based on their mobility across nations. Land and natural resources are fixed in nature. Moreover, wages and capital usage prices are uniform across nations while rent on land and natural resources vary across different industries. Thus, capital accumulation, investment and the labor market would be studied to understand the capitalist economies' economic change. So the entire study is based on the idea that as the economic disruption is propelled by the events like epidemics and, in the current case, a pandemic, the dynamics of the investment and capital market, the labor market and the general wage levels, as well as the rate of inflation, are affected, which cause a cumulative change in the rate of economic growth in the economy. This issue can be understood better with a functional representation of the impact of economic and environmental shocks on the economy.

1. Capital Accumulation and Investment

Under the perfectly competitive markets, the capital stock of a nation at a particular period can be denoted as:

(1) $$\begin{array}{r} K_{r}^{t+1}=K_{r}^{t}+I_{r}^{t}-D_{r}^{t} \end{array}$$

Where $K_{r}^{t+1}$ represents the stock of capital accumulated in an economy r in a given year. $K_{r}^{t}$ is the available capital in region r in year t, $I_{r}^{t}$ is the investment in new capital in region r and year t and $D_{r}^{t}\;$is the depreciation on the capital of region r in year t. This functional relationship allows us to decipher how capital accumulation is achieved in the economy. Next, we move on to the labor markets.

2) The Labor Supply

We start by examining the supply of labor in the labor market of the economy, which is denoted by the following equation:

(2) $$\begin{array}{r} \frac{LS_{r}^{t}}{Pop_{r}^{t}}={\left(RW_{r}^{t}\right)}^{\beta }A_{r}^{t} \end{array}$$

Where, $LS_{r}^{t}$ can be described as the supply of labor in an economy in the period t and region r. $Pop_{r}^{t}$ Denotes the population share in year t and region r. On the right-hand side is the variable ${\left(RW_{r}^{t}\right)}^{\beta }A_{r}^{t}$ which denotes the real post-tax wage of labor type l in year t and region r with $A_{r}^{t}$ and β being the positive constants determining the labor supply in the economy (1).

As the pandemics and epidemics hit the global economy, the death rates escalate, and the population and the pool of available labor resources dwindle in the economy. In turn, it causes a deterioration of the post-tax wage of labor in the economy, which is a further decline in the demand for labor as the economies come to a slow down. We consider the nature and the extent of the impact of the pandemics and epidemics on the economic capital usage and labor supply. The economic implications would be assessed using growth variables such as GDP, investment, consumption pattern, and the wage rates would be examined in the economies while studying the changes emerging along the length of the disease spread of SARS, which stretched from the first quarter of 2002 till the last quarter of 2004, the H1N1 swine flu which stretched from the first quarter of 2009 till the last quarter of 2010 and lastly the current pandemic. The past 2 years' quarterly economic data will be assessed for CME and LME economies to examine the difference in economic growth pre- and post-pandemic times and CME (Japan, Sweden, Germany) and LME (United Kingdom, Australia, Canada, and New Zealand) nations. The study variables' data collection sources include the IMF, World Bank, and OECD database, and the ILOStats. The analysis period has been split across the periods that marked the heart of the disease outbreak, undertaken as a panel data spanning across disrupted timelines and two groups of nations. As part of the research objectives, the qualitative analysis conducted includes studying secondary data to understand capitalist economies' business history during pandemic times. Impact of various pandemic conditions such as SARS (Cov-1 and Cov-2), Influenza H1N1, or the swine flu and the current case of Covid-19 on the economic growth of capitalist nations (comparative analysis of CME and LME nations) has been examined, and recommendations for financial recovery have been made.

## Empirical Findings

We start the analysis process by explaining the exact impact that the recent most disease outbreak, associated with that of the SARS virus and the H1N1 virus, had on the economies under consideration. The analysis then veers off to assess the COVID 19 virus outbreak's impact on the global economy so far. The key variables taken as a proxy for the impact of the disease outbreaks include the hours worked in a week, the rate of unemployment, the inflation rate, the government's investments, and the variable of economic growth.

### Impact of the SARS Outbreak

**Figure 1:**
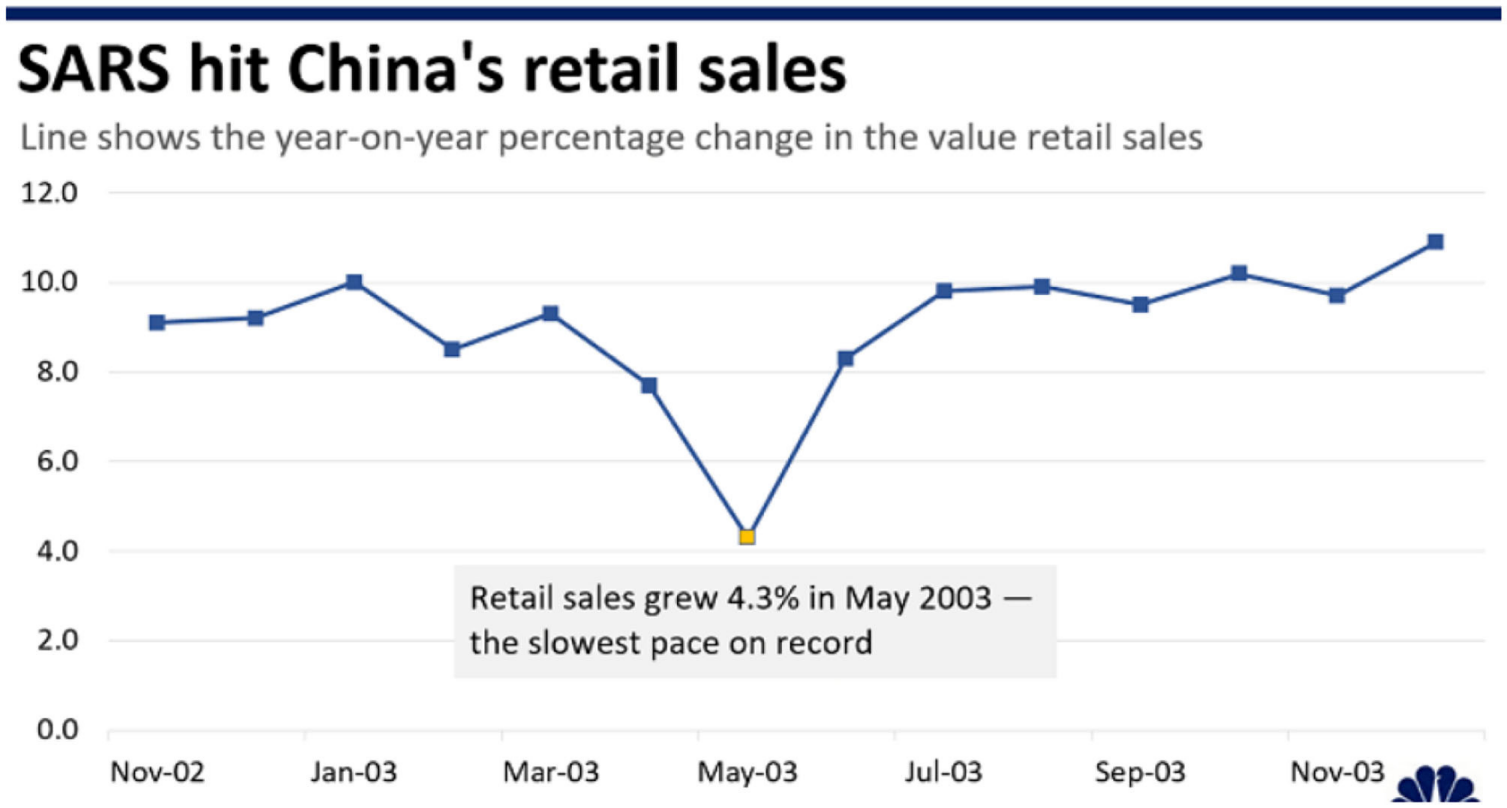
SARS hit China's retail sales [Source (23)].

**Figure 2:**
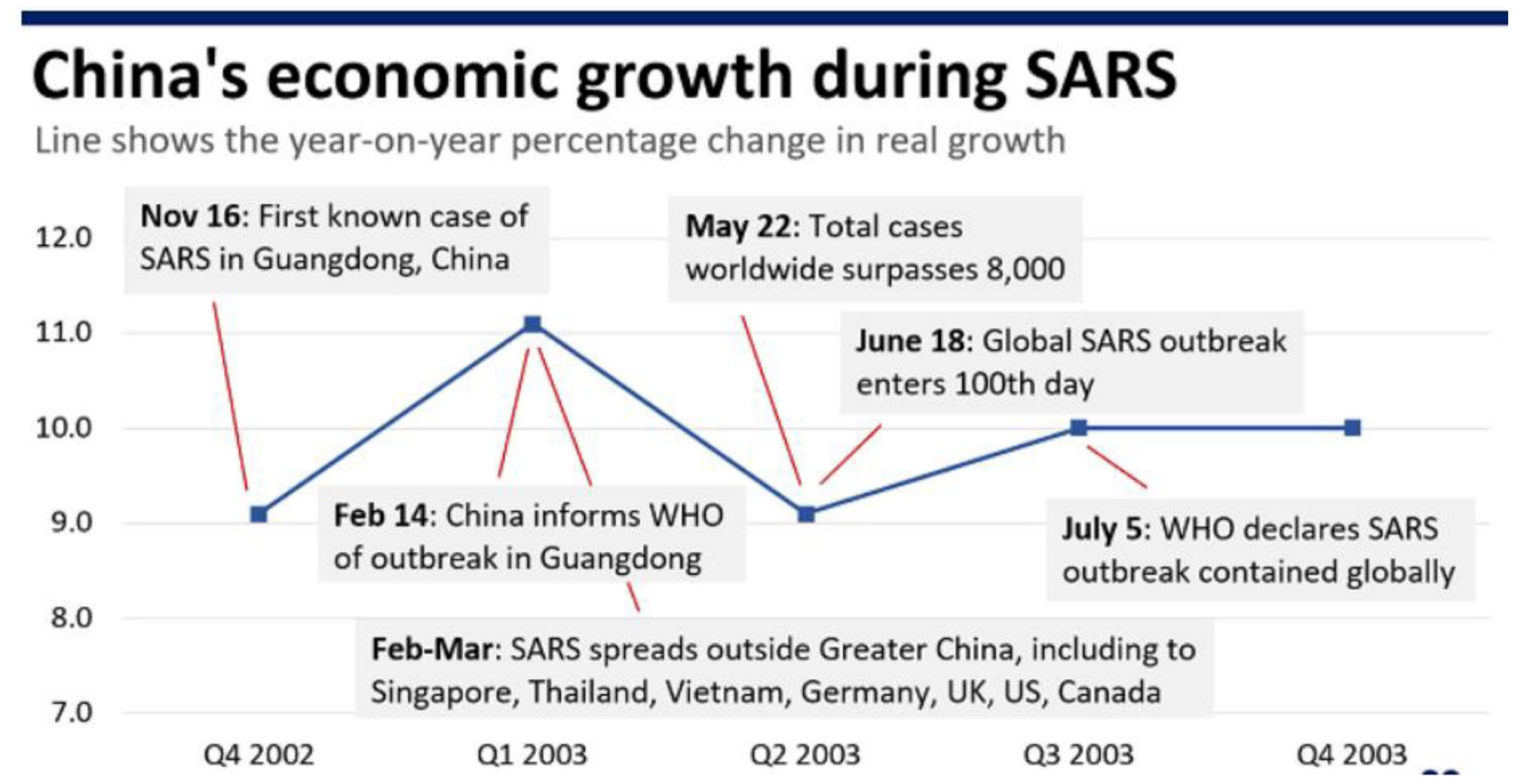
China's economic growth during SARS [Source (23)].

The next section seeks to distinguish the impact of changes in the economy's various sectors and facets, as triggered by the disease outbreak on the economic growth of the coordinated market economies and liberal market economies. Figure 1 indicates that Severe Acute Respiratory Syndrome (SARS) is a viral respiratory disease that is contagious among humans. The disease emerged from the Guangdong province of China in 2002 and was infectious as a cold virus, as shown in Figure 2. It was transmitted to countries like South Africa, Hong Kong, Canada, Australia, Brazil, Spain, and the USA and was contained by July 2003. Approximately 10,000 people were infected, out of which 10% died, and the impact of SARS was devastating on the infected people's health (16). SARS also had an economic impact that became a global concern as major industries involving the gathering of people in public places such as restaurants, travel and tourism, entertainment, and retail establishments.

Various estimations and models anticipated the impact of SARS and the analysis reflected that the influence of SARS on the economies was catastrophic, especially in east Asian and Canadian economies. SARS had a major impact on the investment, retail and tourism industries of China and Hong Kong, making them the most affected areas. China and Hong Kong experienced a significant death toll as well as large short-term economic losses.

**Figure 3:**
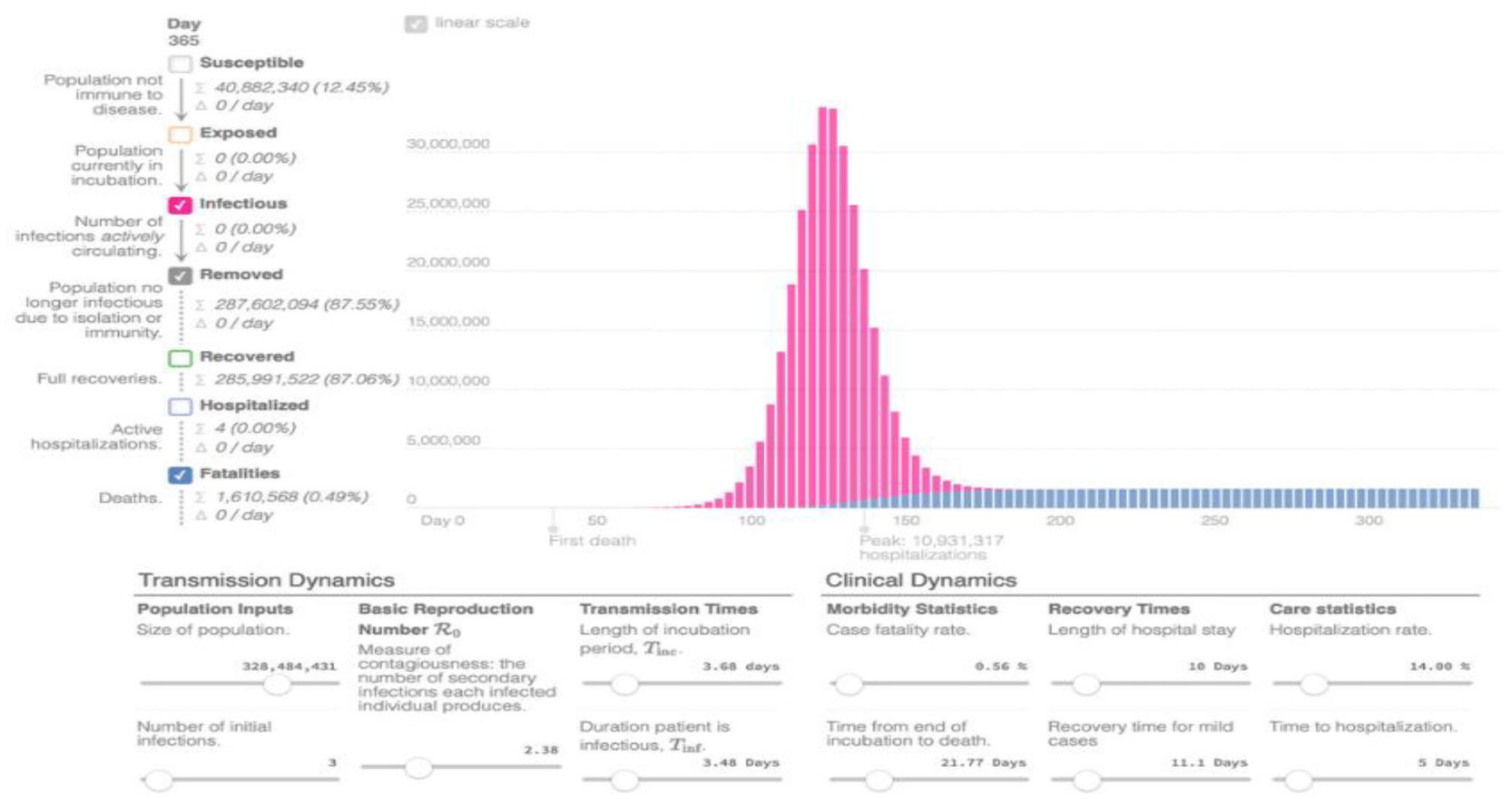
Graph of cases projected in response to the influence model [Source (24)].

These losses corresponded to a short time after which the consumer confidence was restored and many stocks were replenished. The economic consequences of SARS in terms of health expenditure and demographic impacts are small compared to epidemics' economic consequences like HIV/AIDS or malaria. The SARS epidemic was declared over within a year (17). The disease SARS' economic consequences have more indirect damages than direct damages in the affected areas and sectors, as shown in Figure 3. This evidence is because the disease spread quickly across the countries, impacting the residents' health, and the economy was also devastated due to trade and financial linkages among the countries. The economic costs include the private and government medical expenses associated with the disease to diagnose and treat the disease, the cost to sterile environments, take preventive measures and invest in basic research. Due to non-working days lost due to illness or mortality/morbidity, the income foregone is also counted as the epidemic's cost. The foregone income is the capitalized value of future earnings, which is lost because of the deaths and illnesses caused by the disease. Apart from the decline in consumer demand, investments in many sectors have also been impacted. The cost of disease prevention is another economic cost. The global economic impact is enormous because of the transmission of the disease.

We first examine the impact of the SARS outbreak on the UK, Australia, Canada, and New Zealand economies, which can be seen in the first panel of Table 1. Here as is evident, the regression model had an r-squared coefficient of 0.6364, which implies that ~63.64% of the changes in the dependent variable, economic growth, are explained by the changes in the other macroeconomic variables under consideration. Further, it can be noted that in the case of the LMEs that the rate of unemployment and rate of inflation negatively impacted the economic growth. In contrast, the labor force's average hours and the government's investments positively impacted the level of economic growth. However, changes if we move from a fixed-effects model to a random-effects model of economic growth wherein the average hours worked in a week can be seen to have a negative relationship with the level of economic growth. Based on the Hausman test results, which is performed for the null hypothesis that the random-effects model results are more suitable, it can be deciphered that since the test result is statistically significant, we chose the fixed effects model 95% confidence.

**Table 1:** SARS (2002 Q1–2004 Q4): LME vs. CME Nations.

	**LME**		**CME**	
	**Fixed effect**	**Random effects**	**Fixed effect**	**Random effects**
Hours worked in a week	0.0224749	**−0.148513**	0.2416416	0.1001597
	[0.1259515]	[0.0495946]	[0.2235569]	[0.2049271]
Unemployment rate	−0.0556879	−0.0595368	−0.0046404	0.0715892
	[0.091426]	[0.0773679]	[0.085952]	[0.068064]
Inflation rate (CPI)	−0.1355335	−0.1040501	−0.0962139	−0.0392919
	[0.108938]	[0.083778]	[0.2129351]	[0.2152652]
Investment GFCF	0.030037	0.1673616	0.1673616	0.175525
	[0.024467]	[0.0239144]	[0.1108818]	[0.1140778]
Constant	0.7112889	**6.755407**	−8.294725	−4.333864
	[4.470376]	[2.569059]	[8.511295]	[8.260209]
R squared	0.6364	0.8224	0.2718	0.2034
Hausman test statistic	**17.04**		1.94	

*Tables in BOLD reflect statistical significance at 95% level of significance*.

Likewise, the second panel reflects the same model constructed in the CMEs, Japan, Germany, and Sweden to gauge the SARS outbreak's impact. It starts with the r-squared coefficient of 0.2718, reflecting that 27.18% of the dependent variable changes, economic growth, are explained by the changes in the other macroeconomic variables under consideration. Further, it can be noted that in the case of the CMEs, the rate of unemployment and rate of inflation negatively impacted the economic growth. In contrast, the labor force's average hours and the government's investments positively impacted the level of economic growth. However, changed in the random-effects model for the CMEs wherein only the inflation rate negatively impacted economic growth. Based on the Hausman test results, the test estimate is statistically insignificant, making the random effects model more suitable for the CMEs.

### Impact of the H1N1 Swine Flu Outbreak

**Figure 4:**
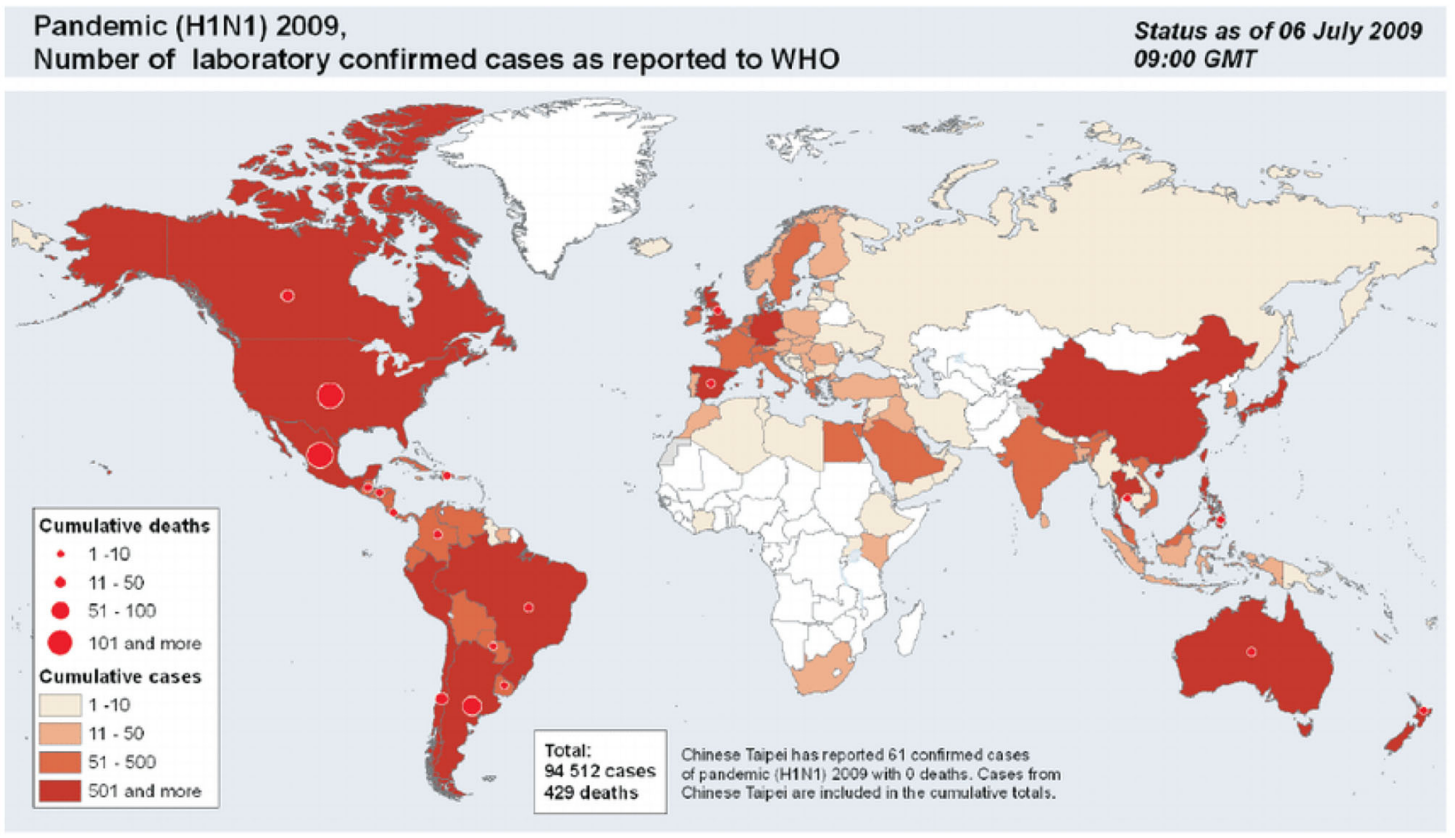
Graph showing the impact of H1N1 pandemic all over the world.

In his book “Against Empire,” Michael Parenti says that “The essence of capitalism is to turn nature into commodities and commodities into capital. The live green earth is transformed into dead gold bricks, with luxury items for the few and toxic slag heaps for the many.” As we know now, the world has evolved with each pandemic or invention it had to face, good or bad, as shown in Figure 4. From time immemorial, disasters like the Great Fire of London in 1666, the Galveston hurricane, the sinking of the Titanic in 1912 and diseases like the Bubonic plague and very recently the Coronavirus pandemic, all have had their fair share of impact on the capitalistic economy (18).

It is no jargon when one says that a pandemic can leave a nation extremely handicapped and stripped raw, especially when observed in industry evaluation and equity analysis. Speaking specifically in terms of the H1N1 Swine Flu, which was caused by a strain of the influenza virus commonly found in pigs and having symptoms mirroring influenza, the disease saw a huge economic recession post it's spread, which entailed a severe crash in the stock market values of industries, crashing established policies, to name a few. The idea behind smart investments, like that in Gold, was then tested. Despite thinking tanks at work constantly designing mathematical models that predict and theories that considered almost all the permutations and combinations of the possible worst-case scenarios, some economic shocks surfaced. The following are the effect of H1N1 on a capitalistic economy.

- There was a surge in demand for hospital and other medical services.
- There was a temporary upsurge in sick leave and school closures requiring the withdrawal of working-class parents.
- There were unprecedented deaths with a corresponding permanent reduction in the labor force.
- There was a cap on international tourism and business travel.
- Poverty in developing counties and the quality of health care systems in these economies make it harder for them to recover from big losses (19).

From this, will it be safe to conclude that outbreaks such as these break the system irrevocably, and nothing can be done about it? Not. A quick observation will not leave us privy to the fact that these pandemics have strengthened the global health system by urging the authorities in many countries to develop pandemic response plans, each more responsive and flexible than the last, an idea which has successfully been backed by WHO. Although these pandemics come as a massive shock initially and are extremely significant, all economies must understand that it is over relatively quickly if all the forces above come into play.

The current section presents the differences between the impact of changes in the various sectors and facets of the economy, as triggered by the disease outbreak on the economic growth of the coordinated market economies and liberal market economies. We first examine the impact of the H1N1 outbreak on the UK, Australia, Canada, and New Zealand economies, which can be seen in the first panel of Table 2. Here the goodness of fit of the regression model can be interpreted through its r-squared coefficient of 0.6485, which implies that ~64.85% of the changes in the dependent variable, economic growth, are explained by the changes the other macroeconomic variables as caused by the spread of H1N1 virus. Further, it can be noted that in the case of the LMEs that the rate of unemployment negatively impacted the economic growth. In contrast, the labor force's average hours and the government's investments positively impacted the economic growth level—the nature of the random-effects model's relationship. Based on the Hausman test results, it can be deciphered that since the test result is statistically significant, we chose the fixed effects model with 95% confidence.

**Table 2:** H1N1 Swine Flu (2009 Q1–2010 Q4): LME vs. CME Nations.

	**LME**		**CME**	
	**Fixed effect**	**Random effects**	**Fixed effect**	**Random effects**
Hours worked in a week	**0.6525314**	**0.0553695**	**0.6543426**	**0.4723728**
	[0.254869]	[0.0761379]	[0.2675615]	[0.1857754]
Unemployment rate	**−0.2074054**	**−0.2199893**	0.0485426	**0.1136841**
	[0.05994]	[0.0574449]	[0.1252462]	[0.0419675]
Inflation rate (CPI)	**0.4279377**	**0.4722499**	0.1117892	0.3471743
	[0.214858]	[0.209494]	[0.384277]	[0.3097005]
Investment GFCF	**0.3413916**	**0.3655099**	**0.4214208**	**0.431975**
	[0.037242]	[0.0371364]	[0.1087446]	[0.1052326]
Constant	**−21.16356**	−0.1264334	**−24.46609**	**−18.8801**
	[9.12589]	[2.907771]	[9.030853]	[7.163458]
R squared	0.6485	0.4107	0.325	0.7187
Hausman test statistic	**13.26**		1.34	

*Tables in BOLD reflect statistical significance at 95% level of significance*.

Likewise, the second panel reflects the same model constructed in the context of the CMEs, Japan, Germany, and Sweden to gauge the impact of the H1N1 outbreak. In the case of the CMEs, the independent variables, including unemployment rate, weekly hours worked, inflation rate and investments made by the government, positively impacted the economic growth during the H1N1 virus outbreak. This evidence did not change in the random-effects model for the CMEs, which held similar relationships. Based on the Hausman test results, the test estimate is statistically insignificant, making the random effects model more suitable for the CMEs.

We now move forth to examining the nature of issues being faced due to the COVID 19 crisis.

### Impact of the COVID 19 Outbreak

Covid-19 has impacted the societies in far more ways than impacting the health of the affected. It is affecting the societies as well as the economies at the core. The impact of the pandemic is severe and vary from country to country. It is likely to increase the economic costs among nations and increase the inequalities at a global level (20). The pandemic has disrupted the lives of people and affected world trade and movements, as seen in Table 3. At this stage, the pandemics negatively affect the manufacturing sector. Various industries and sectors have slowed down because of the disease, such as tourism, pharmaceutical industry, solar power sector, information, and electronics industry. There have been short-term challenges like a halt in tourism and entertainment and long-term consequences such as disruptions in trade and investments (21). The disease has extensive consequences on the healthcare, economic, and social sector.

**Table 3:** COVID 19 (2019 Q1–2020 Q2): LME vs. CME Nations.

	**LME**		**CME**	
	**Fixed effect**	**Random effects**	**Fixed effect**	**Random effects**
Hours worked in a week	**1.27869**	**−0.0104511**	**1.401871**	**1.103879**
	[0.369166]	[0.0701911]	[0.434022]	[0.3162875]
Unemployment rate	**−0.6886533**	**−0.8512298**	−0.0284523	0.0332361
	[0.0594945]	[0.0685536]	[0.3330152]	[0.0425666]
Inflation rate (CPI)	**0.4554238**	0.3974084	1.846953	**2.58637**
	[0.2853351]	[0.3771379]	[1.253874]	[0.7055796]
Investment GFCF	**0.3532495**	**0.3952025**	**1.088862**	**1.024693**
	[0.0212801]	[0.0279858]	[0.1760394]	[0.1625048]
Constant	**−37.37288**	**9.344054**	**−52.10929**	**−42.97107**
	[13.10738]	[2.968868]	[16.9909]	[11.94268]
R squared	0.3243	0.9154	0.0419	0.9357
Hausman test statistic	**10.59**		1.3	

*Tables in BOLD reflect statistical significance at 95% level of significance*.

#### Healthcare Impact

The healthcare sector faces challenges in the pandemic regarding diagnosis, treatment, and disease prevention. The medical system's functioning has become a burden, and patients with other medical problems are getting neglected. The lives of doctors and other health professionals are at very high risk. Pharmaceutical shops are overloaded, and the medical supply chain is disrupted.

#### Economic Impact

Due to the lockdown and the risk of spreading the disease, the manufacturing of essential goods has slowed down. The supply chain of products has been disrupted, and national and international businesses face losses (22). The cash flow in the market is poor, slowing down the revenue growth in the economy. Millions of workers have lost their jobs as industries have shut down. The GDP of many economies have also been impacted due to production in industries being disrupted.

#### Social Impact

The society has been impacted in a lot of ways. The service sector has not been able to serve people due to the unavailability of products. Large-scale events and sports tournaments have been postponed or canceled to avoid public gatherings. National and international traveling has been banned, and cultural and religious events have also been disrupted. There has been witnessed undue stress among people as they have to maintain social distancing from peers, family, and friends. The closure of hotels, restaurants, and cinemas has also disrupted the lives of people. The education industry has also been impacted in many ways, such as postponement of examinations and class cancellation.

**Figure 5:**
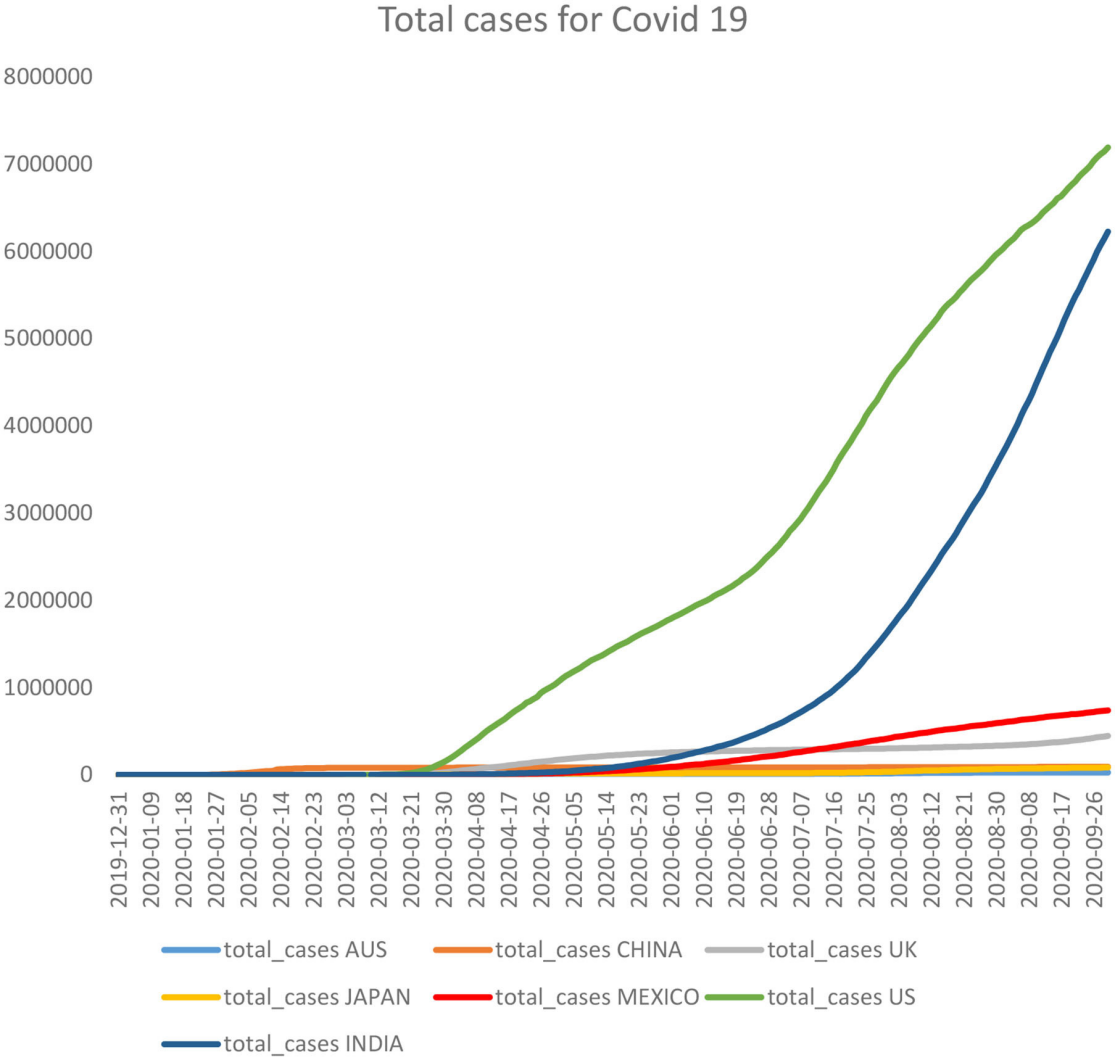
Number of deaths caused by COVID-19 (Source: self-generated).

As can be seen from Figure 5, the incidence of deaths caused by COVID-19 increased monumentally since the onset of the second quarter of 2020, which forced the governments to venture forth with the idea of nationwide lockdowns.

We examine the nature of the relationship between macroeconomic variables and the economic growth triggered by the Coronavirus outbreak. Here, it can be noted that in the case of the LMEs that the rate of unemployment negatively impacted the economic growth. In contrast, the labor force's average hours, rate of inflation and the government's investments positively impacted economic growth. However, if we move from a fixed-effects model to a random-effects economic growth model wherein the average hours worked in a week can negatively affect economic growth and the unemployment rate. Based on the Hausman test results, it can be deciphered that since the test result is statistically significant, we chose the fixed effects model with 95% confidence. In the case of the impact of coronavirus spread in the CME economies, every macroeconomic variable, including unemployment, average hours worked, inflation rate, and government investments, positively impacted economic growth. This evidence is changed in the random-effects model for the CMEs wherein only the inflation rate negatively impacted economic growth. Based on the Hausman test results, the test estimate is statistically insignificant, making the random effects model more suitable for the CMEs.

Thus, it can be deciphered that considerable differences exist in how the economies are affected by the disease outbreaks. The LME economies, including the UK, Canada, and Australia sample, reflected that the unemployment rate and inflation rate negatively impacted economic growth. In contrast, government investment positively impacted the economic growth, as was expected. On the other hand, in the CME economies, the variables all positively related to the economy's economic growth. This evidence can be explained by the intrinsic nature of the coordinated market economies wherein the issues faced by the businesses are resolved by the government institutions (25). Thus, as the governments control the macro variables, the shocks presented by the events like disease outbreaks do not impact the nation's economic growth. This finding explains the positive relationship between the macroeconomic variables and economic growth. This issue is different in the case of the Liberal market economies, wherein the market forces act together to determine the macro variables' flow.

## Conclusion

As the disease outbreaks occur, they stand to impact various facets of the economy, including the capital markets, labor markets, foreign trades, and the consumption and production sectors. The current study sought to examine the impact of the disease outbreaks like the current coronavirus pandemic on the economy, differentiated by the varieties of capitalist structures. The data analysis included the assessment of the impact of the SARS virus, H1N1 virus and the COVID19 virus, computed in the context of the coordinated market economies of Germany, Sweden, and Japan and liberal market economies of Australia, New Zealand, the United Kingdom, and Australia. The analysis revealed that the LME economies were more responsive to the impact of the disease outbreaks than the CME economies, wherein the government involvement moderated the disease's impact. This evidence allows us to conclude that increasing government authorities' involvement can moderate the disease outbreaks' impact.

## Data Availability Statement

The original contributions presented in the study are included in the article/supplementary material, further inquiries can be directed to the corresponding author.

## Author Contributions

YS: conceptualization, methodology, formal analysis, writing—original draft preparation, project management, and funding acquisition. HL: conceptualization, methodology, writing—original draft preparation, and funding acquisition. RZ: methodology, writing—review, and editing. All authors have read and agreed to the published version of the manuscript.

## Conflict of Interest

The authors declare that the research was conducted in the absence of any commercial or financial relationships that could be construed as a potential conflict of interest. The reviewer XW declared a shared affiliation, though no other collaboration, with one of the authors HL to the handling Editor.

## References

[1] Verikios G. The dynamic effects of infectious disease outbreaks: the case of pandemic influenza and human coronavirus. Socio Econ Plann Sci. (2020) 71:100898. 10.1016/j.seps.2020.100898 32834133 PMC7286241

[2] World Health Organization. The world health report 2004: changing history. J Interprof Care. (2004) 21:1–2.

[3] Henderson R. Reimagining capitalism in the shadow of the pandemic. Harvard Business Review. (2020). Available online at: https://hbr.org/2020/07/reimagining-capitalism-in-the-shadow-of-the-pandemic (accessed September 26, 2020).

[4] French N. How capitalism kills during a pandemic. Jacobin. (2020). Available online at: https://www.jacobinmag.com/2020/03/capitalism-pandemic-coronavirus-covid-19-single-payer (accessed September 26, 2020).

[5] Perry J Sayndee TD. Social Mobilization and the Ebola Virus Disease in Liberia. Rowman & Littlefield (2016).

[6] Thelen K. Varieties of capitalism and business history. Business Hist Rev. (2010) 84:646–8. 10.2307/27917300

[7] Huber E Petrova B Stephens JD. Financialization and Inequality in Coordinated and Liberal Market Economies. LIS Working papers (2018).

[8] McLaughlin C Wright CF. The role of ideas in understanding industrial relations policy change in liberal market economies. Indus Relat J Econ Soc. (2018) 57:568–610. 10.1111/irel.12218

[9] Horner R. Towards a new paradigm of global development? Beyond the limits of international development. Progress Hum Geogr. (2020) 44:415–36. 10.1177/0309132519836158

[10] Abrams EM Szefler SJ. COVID-19 and the impact of social determinants of health. Lancet Respir Med. (2020) 8:659–61. 10.1016/S2213-2600(20)30234-4 32437646 PMC7234789

[11] Anner M. Abandoned? The Impact of Covid-19 on Workers and Businesses at the Bottom of Global Garment Supply Chains. (2020).

[12] Barrientos S. Gender and Work in Global Value Chains: Capturing the Gains? Cambridge: Cambridge University Press (2019).

[13] Brooks R Ribakova E Lanau S Fortun J Hilgenstock B. Capital Flows Report: Sudden Stop in Emerging Markets. Washington, DC: Institute of International Finance (2020). Available online at: https://www.iif.com/Portals/0/Files/content/2_IIF2020_April_CFR.pdf (accessed September 18, 2020).

[14] Kentikelenis A Gabor D Ortiz I Stubbs T McKee M Stuckler D. Softening the blow of the pandemic: will the International Monetary Fund and World Bank make things worse? Lancet Global Health. (2020) 8:e758–9. 10.1016/S2214-109X(20)30135-2 32278363 PMC7146698

[15] Huang Y Sun M Sui Y. How digital contact tracing slowed Covid-19 in East Asia. Harvard Business Review. (2020). Available online at: https://hbr.org/2020/04/how-digital-contact-tracing-slowed-covid-19-in-east-asia (accessed September 18, 2020).

[16] Keogh-Brown MR Smith RD. The economic impact of SARS: how does the reality match the predictions? Health Policy. (2008) 88:110–20. 10.1016/j.healthpol.2008.03.003 18436332 PMC7114672

[17] Lee JW McKibbin WJ. Estimating the global economic costs of SARS. In: Learning From SARS: Preparing for the Next Disease Outbreak: Workshop Summary. Washington, DC: National Academies Press (2004). p. 92–109. 22553895

[18] Verikios G Sullivan M Stojanovski P Giesecke J Woo G. The Global Economic Effects of Pandemic Influenza. (2011).

[19] Mckibbin W. The Swine Flu Outbreak and Its Global Economic Impact. (2009). Available online at: https://www.brookings.edu/on-the-record/the-swine-flu-outbreak-and-its-global-economic-impact/ (accessed 9 May 2020).

[20] Sumner A Hoy C Ortiz-Juarez E. Estimates of the Impact of COVID-19 on Global Poverty. WIDER Working Paper Series (2020).

[21] Walker P Whittaker C Watson O Baguelin M Winskill P Hamlet A. The impact of COVID-19 and strategies for mitigation and suppression in low- And middle-income countries. Science. (2020) 369:eabc0035. 10.1126/science.abc0035 32532802 PMC7292504

[22] Bartik AW Bertrand M Cullen Z Glaeser EL Luca M Stanton C. The impact of COVID-19 on small business outcomes and expectations. Proc Natl Acad Sci USA. (2020) 117:17656–66. 10.1073/pnas.2006991117 32651281 PMC7395529

[23] Lee Y. 4 charts show how SARS hit China's economy nearly 20 years ago. China Economy. (2020). Available online at: https://www.cnbc.com/2020/02/11/coronavirus-4-charts-show-how-sars-hit-chinas-economy-in-2003.html (accessed September 4, 2020).

[24] Rossman J. Coronavirus: What the 2009 Swine Flu Pandemic Can Tell Us About the Weeks to Come. (2020). Available online at: https://theconversation.com/coronavirus-what-the-2009-swine-flu-pandemic-can-tell-us-about-the-weeks-to-come-134076 (accessed October 24, 2020).

[25] Ebner A. Varieties of capitalism and the limits of entrepreneurship policy: institutional reform in Germany's coordinated market economy. J Indus Competition Trade. (2010) 10:319–41. 10.1007/s10842-010-0086-x

